# Protective fibroblastic niches in secondary lymphoid organs

**DOI:** 10.1084/jem.20221220

**Published:** 2023-12-01

**Authors:** Angelina De Martin, Yves Stanossek, Natalia Barbara Pikor, Burkhard Ludewig

**Affiliations:** 1 Institute of Immunobiology, Medical Research Center, Kantonsspital St.Gallen, St.Gallen, Switzerland; 2 Department of Otorhinolaryngology, Head and Neck Surgery, Kantonsspital St.Gallen, St.Gallen, Switzerland; 3 Institute of Microbiology, ETH Zurich, Zurich, Switzerland

## Abstract

Fibroblastic reticular cells (FRCs) are specialized fibroblasts of secondary lymphoid organs that provide the structural foundation of the tissue. Moreover, FRCs guide immune cells to dedicated microenvironmental niches where they provide lymphocytes and myeloid cells with homeostatic growth and differentiation factors. Inflammatory processes, including infection with pathogens, induce rapid morphological and functional adaptations that are critical for the priming and regulation of protective immune responses. However, adverse FRC reprogramming can promote immunopathological tissue damage during infection and autoimmune conditions and subvert antitumor immune responses. Here, we review recent findings on molecular pathways that regulate FRC–immune cell crosstalk in specialized niches during the generation of protective immune responses in the course of pathogen encounters. In addition, we discuss how FRCs integrate immune cell–derived signals to ensure protective immunity during infection and how therapies for inflammatory diseases and cancer can be developed through improved understanding of FRC–immune cell interactions.

## Teaser

Lymphoid organ fibroblasts, known as fibroblastic reticular cells (FRCs), generate specialized niches in the tissue and coordinate immune cell interactions. De Martin and colleagues discuss basic concepts of FRC biology and how FRCs determine activation or attenuation of immune responses.

## Structural foundations of protective immunity in SLOs

The functionality of multicellular tissues in the mammalian organism depends on the sustenance of homeostatic circuits that regulate cellular and molecular interactions within physiological concentrations and rates (Meizlish et al., 2021). Inflammatory perturbation, for example, during infection, leads to loss of tissue structure and function and to overriding of homeostatic control mechanisms (Medzhitov, 2021). Thus, protective immunity includes all immunological pathways, components, and structures that ensure the maintenance of tissue integrity and function. One of the main challenges for the establishment of protective immunity in the mammalian immune system is scaling of immune processes across species with largely different body sizes, e.g., mice versus humans versus elephants. Such scaling of protective T cell and B cell responses is best explained by the concept of a basic functional unit, the protecton (Langman and Cohn, 1987). Cohn and Langman suggested that about 10^7^ B cells constitute such an evolutionarily selectable and functional unit covering 5 × 10^4^ specificities (Cohn and Langman, 1990). Indeed, although the sequence diversity of unique B cell receptors based on the usage of different combinations of V, (D), and J germline-encoded genes and additional processes is around 10^15^ in human naive B cells (Briney et al., 2019), functional diversity of antibodies is significantly lower due to epitope promiscuity and polyreactivity (Rees, 2020). Likewise, whereas VDJ recombination in T cells can generate a diversity of 10^14^ different sequences in the variable CDR3 region of the human TCRβ chain (Murugan et al., 2012), only 1 per 10^4^–10^6^ T cells recognize a specific epitope (Jenkins and Moon, 2012). Thus, the specific microenvironments that support activation of protective units of T and B cells in secondary lymphoid organs (SLOs) have to be scaled across mammalian species in a fashion that generates repetitive structural units that surveil roughly similar volumes of bodily tissues.

Three major classes of SLOs support immune surveillance in the mammalian organism: the splenic white pulp, lymph nodes (LNs), and Peyer’s patches (Junt et al., 2008). SLOs are built from standardized functional building blocks to perform their key function of filtering and immunologically screening extracellular fluids, with LNs collecting lymph, the white pulp of the spleen clearing the blood of immunologically harmful material, and Peyer’s patches constantly probing the fluids of intestinal contents to build anti-commensal and anti-pathogen immunity (Acton et al., 2021). The basic SLO structure is tailored to facilitate coordinated communication and efficient activation of immune cells within specialized niches such as the antigen sampling zone, B cell follicles, and the T cell zone (Junt et al., 2008). Conserved immune cell interactions including activation of T cells by dendritic cells (DCs) in the T cell zone (Castellino et al., 2006), crosstalk between T and B cells at the T–B border (Roco et al., 2019), or refinement of antibody specificity during the germinal center (GC) reaction in the B cell zone (Pikor et al., 2020) are coordinated in spatially defined niches. Moreover, conservation of entry and migration channels secure in-and-out immune cell trafficking in SLOs and transition of immune cells between specific niches. A common entry route for naive lymphocytes is provided by specialized post-capillary venules, termed high-endothelial venules (HEVs), in LNs and Peyer’s patches (Ager, 2017), while activated lymphocytes can enter LNs or the splenic white pulp through the antigen sampling zone (Lewis et al., 2019; Thomas et al., 2016). Importantly, specialized fibroblast populations, known as fibroblastic reticular cells (FRCs), generate functionally equivalent environments in SLOs to ensure the productive interaction of immune cells for the establishment of protective immunity.

In this review, we present basic concepts of FRC biology and discuss recent findings on how FRC-derived molecules determine activation or attenuation of immune responses. In particular, we focus on FRC–immune cell interactions in specialized niches during the generation of protective immune responses in the course of pathogen encounters. Knowledge on particular niche functions of FRCs during homeostasis and infection will be instrumental to guide the development of therapies for inflammatory diseases and cancer.

## FRC ontogeny and differentiation

The core functions of FRCs for establishing and maintaining dedicated niche environments in SLOs include (i) the generation of the fibrous network of extracellular matrix (ECM) components and the conduit system that funnels liquids and low-molecular-weight compounds through B and T cell zones (Novkovic et al., 2020), (ii) the production of constitutive chemokines such as CXCL12, CXCL13, CCL19, and CCL21 for migration and positioning of immune cells (Schulz et al., 2016), and (iii) provision of growth factors, co-stimulatory molecules, cytokines, and alarmins that secure immune cell homeostasis in the particular niche and secure appropriate differentiation during immune activation (Lütge et al., 2023). The diverse functions and phenotypes of FRC subsets are determined by their developmental origin and are further shaped by their position within the SLOs and functional status of interacting immune cells.

### Developmental origin of FRCs

The development of LNs and Peyer’s patches is determined by the influx of lymphoid tissue inducer cells, which accumulate at the respective anlagen (Bovay et al., 2018; Onder et al., 2017; Prados et al., 2021) and initiate the activation of endothelial lymphoid tissue organizer (LTo) cells (Bovay et al., 2018; Onder et al., 2017). Although it has been suggested that LN development relies on retinoic acid–mediated neuronal activation of mesenchymal LTo cells (van de Pavert and Mebius, 2010), recent evidence indicates that distinct transcription factors including odd-skipped related transcription factor 1 determine mesenchymal LTo cell differentiation (Vallecillo-García et al., 2023). Likewise, differentiation of early mesenchymal LTo cells in the splenic primordium is driven by the transcription factors Nkx2–5 and Islet1 (Castagnaro et al., 2013). Although the key transcription factors of mesenchymal LTo cells driving Peyer’s patch development remain largely unexplored, it is evident that tissue origin explains much of the phenotypic differences between FRCs of the major SLOs (Lütge et al., 2023). Importantly, additional signals derived from immune cells are of key importance for the final shaping of fibroblastic niches in SLOs (Lütge et al., 2021).

The initiation of FRC lineage commitment and niche maturation in all SLOs is mediated via the lymphotoxin β-receptor (LTβR) and the NF-κB pathway (Lütge et al., 2021). Notably, LTβR-dependent signals appear to function as a major switch, i.e., a “signal 1,” to mediate FRC linage commitment of different embryonic FRC progenitor populations (Lütge et al., 2021; Pikor et al., 2021). In the splenic white pulp, FRCs derive from periarterial mesenchymal LTo cells (Cheng et al., 2019), whereas FRCs in Peyer’s patches originate from two distinct subepithelial and perivascular mesenchymal LTo cells (Prados et al., 2021). In the LN anlage, perivenous mesenchymal LTo cells are the most likely progenitor cell population that gives rise to differentiated FRC subsets (Onder et al., 2017). Subsequent FRC differentiation steps with the acquisition of phenotypical traits required for niche formation are driven by additional “second” signals, which include RANK and TNFR1 to support marginal RC (MRC) and follicular DC (FDC) differentiation, respectively (Camara et al., 2019; Prados et al., 2021). Other secondary signals affecting FRC differentiation are integrated through the Hippo pathway, with expression of yes-associated protein (YAP) and transcriptional coactivator with PDZ-binding motif in Ccl19-Cre^+^ cells being critical for FRC commitment and maturation (Choi et al., 2020). While novel insights into FRC ontogeny, subset identity, and function in various murine SLOs have been gained in recent years, our knowledge of human FRC development and functions in homeostasis and disease is still limited.

### FRC subsets in murine and human SLOs

Following the initial description of “reticular cells” underpinning B cell zones in lymphoid organs (Coons et al., 1951), the fibroblastic cells in the GC had been named “dendritic” RCs, mainly based on their morphological appearance (Nossal et al., 1965). A more accurate concept for a consistent terminology of lymphoid organ fibroblasts has been provided by Gretz and colleagues (Gretz et al., 1997). Subsequently, the term “FRC” has been assigned mainly to murine LN fibroblasts of the T cell zone that express the type-I integral membrane glycoprotein podoplanin (PDPN) and lack the endothelial cell marker CD31 (Link et al., 2007). However, this simplified marker-based definition falls short of adequately defining FRC subsets in cross-species comparison (Lütge et al., 2023), with human splenic white pulp fibroblasts completely lacking PDPN expression (Alexandre et al., 2022). Recent single-cell transcriptomics analyses of murine and human lymphoid organ fibroblasts combined with flow cytometric and histological validation strongly support the regional and functional division into different subsets: B cell zone RCs (BRCs) including MRCs in the antigen-sampling zone and FDCs underpinning the GC, T cell zone RCs (TRCs) in the T cell zone, and perivascular RCs (PRCs) that connect the lymphatic and blood endothelial networks with the FRC network (Alexandre et al., 2022; De Martin et al., 2023; Kapoor et al., 2021; Lütge et al., 2023; Perez-Shibayama et al., 2020; Pezoldt et al., 2018; Pikor et al., 2020; Prados et al., 2021; Rodda et al., 2018). The following transcriptomics-based description of the FRC landscape focuses on those subsets whose location and marker combinations have been confirmed by immunohistology or equivalent methods.

**Figure 1.**
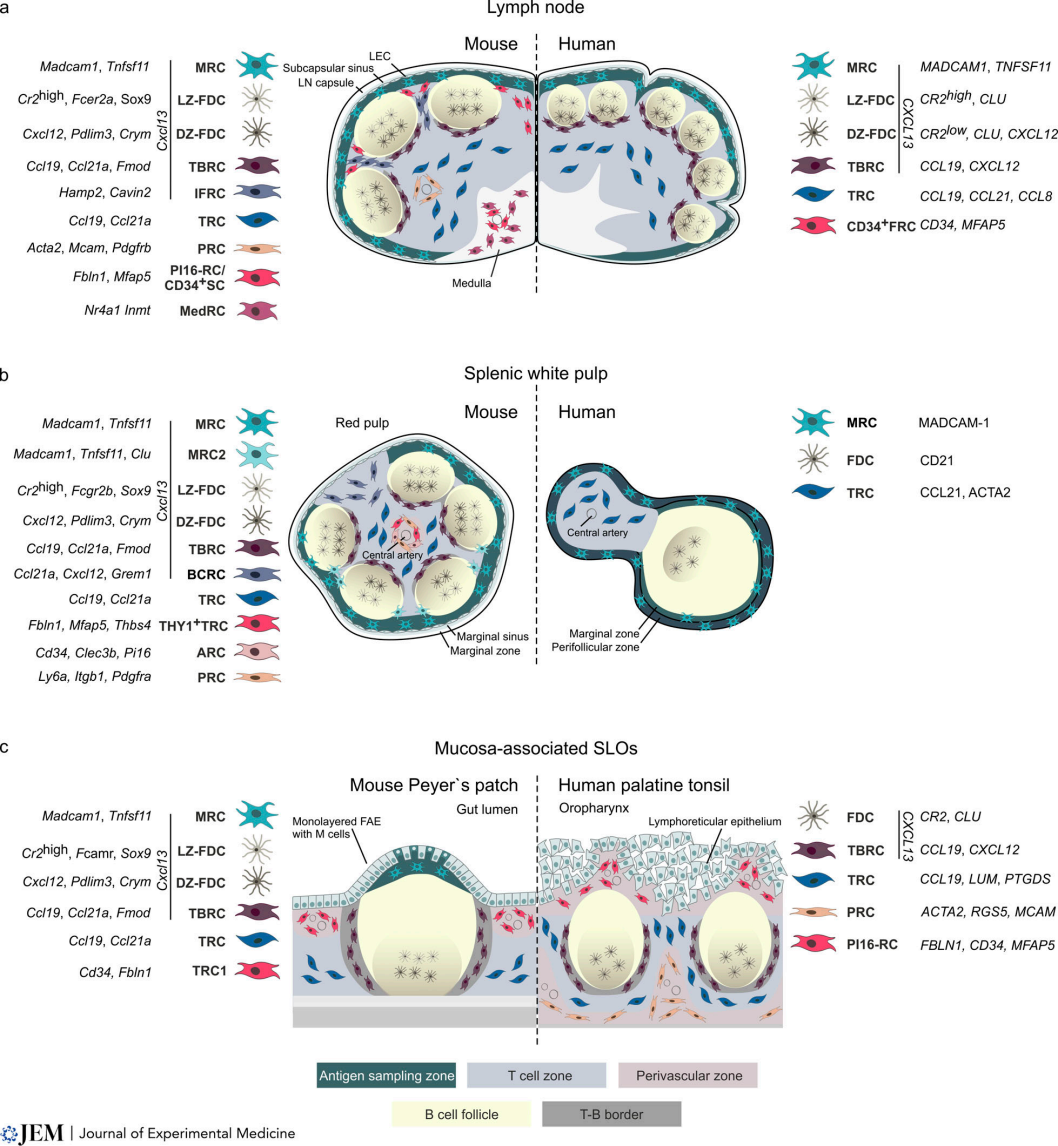
**FRC subsets in murine and human SLOs.** Schematic representation and marker annotation of FRC subsets in relation to the antigen sampling zones, T and B cell zones, and perivascular zones. *Cxcl13*-expressing BRCs include MRCs, FDCs, TBRCs, IFRCs, and BCRCs. MRCs underlie the subcapsular sinus (lymph node), the marginal sinus bordering red pulp, and B cell follicles (spleen) or the subepithelial dome (Peyer’s patch). In the human spleen, MRCs underlie the marginal zone and the perifollicular zone. LZ- and DZ-FDCs form the FDC network in the B cell follicle. TBRCs line the interface of the B cell follicle and T cell zone. TRCs are localized to the T cell zone. **(a)** In lymph nodes, IFRCs support the areas between B cell follicles. PRCs and PI16-RCs or CD34^+^ stromal cells (CD34^+^ SC) support perivascular niches, and MedRCs locate to the medulla. **(b)** In the spleen, BCRCs support bridging channels. THY1^+^ TRCs, ARCs, and PRCs are located in the perivascular space around the central artery. **(c)** Subepithelial perivascular niches are supported by the TRC1 subset in murine Peyer’s patches and PI16-RCs in human palatine tonsils. PRCs support perivascular niches in T cell–rich tonsillar regions. M, microfold; FAE, follicle-associated epithelium; LEC, lymphatic endothelial cell.

MRCs are situated in the antigen-sampling zone, i.e., the subcapsular sinus of LNs (Fig. 1 a), the marginal sinus of the spleen (Fig. 1 b), and the subepithelial dome of Peyer’s patches (Fig. 1 c). Lymph-borne antigens reach LNs via afferent lymphatics that open into the subcapsular sinus, while arterially delivered antigens enter the splenic marginal sinus, where MRCs orchestrate myeloid cell positioning, B cell migration, and further antigen display and processing (Katakai, 2012; Lewis et al., 2019). Across murine SLOs, MRCs appear as a conserved FRC subset characterized by *Cxcl13*, *Madcam-1*, and *Tnfsf11* (TRANCE, RANKL) gene expression (Lütge et al., 2023; Fig. 1, a–c). Single-cell transcriptomics analyses of *CXCL13*-expressing FRCs in human LNs revealed canonical marker gene signatures similar to murine MRCs including *CXCL13*, *MADCAM1*, and *TNFSF11* (Lütge et al., 2023). Although the marginal zone in the human spleen lacks a marginal sinus, histological analyses suggest that the red pulp–white pulp border area harbors MADCAM-1^+^ MRC-like cells (Fig. 1 b, right panel; Alexandre et al., 2022; Steiniger et al., 2014). While in murine mucosa-associated lymphoid tissues surveying the intestinal lumen, MRCs are localized in the subepithelial dome (Prados et al., 2021), MRCs are not present in human palatine tonsils (Fig. 1 c, right panel), as shown by histological staining for TRANCE (RANKL; Mourcin et al., 2021) and extended single-cell transcriptomics analysis (De Martin et al., 2023). It is conceivable that antigen sampling from the oropharyngeal contents is performed by the specialized lymphoreticular epithelium, which forms a meshwork interspersed with lymphocytes and myeloid cells (De Martin et al., 2023).

BRCs, as defined by the expression of *Cxcl1*3 and their location in the B cell follicle, efficiently direct B cell positioning within LNs by altering CXCL13 gradients (Cosgrove et al., 2020). It has been shown that several BRC subsets are conserved across mouse and human SLOs including prototypic FDCs, MRCs, and T–B border RCs (TBRCs; Lütge et al., 2023; Fig. 1, a–c). Single-cell transcriptional profiling of murine LNs has revealed two distinct FDC subsets: light zone (LZ)- and dark zone (DZ)-FDCs (Pikor et al., 2020). LZ-FDCs express the highest levels of *Cr2*, *Fcer2a*, and *Sox9* and are specialized to present antigens in the LZ during the GC reaction. DZ-FDCs express lower levels of genes related to antigen presentation but higher levels of *Cxcl12*, *Pdlim3*, and *Crym* (Lütge et al., 2023; Pikor et al., 2020). Fc receptor gene expression distinguishes LZ-FDCs in spleen and Peyer’s patches by *Fcgr* and *Fcamr* expression, respectively (Alexandre et al., 2022; Lütge et al., 2023). In human LNs and tonsils, CXCL13-expressing BRCs were identified with a similar canonical FDC marker signature including *CR2* and *CLU* genes. Moreover, polarized expression of *CR2*, *FCER2* (LZ-FDC), and *CXCL12* (DZ-FDC) within human LNs implies the separation into LZ- and DZ-FDCs (Lütge et al., 2023). Topological and in-depth molecular definition of histologically detected human splenic CD21^+^ FDCs is still lacking (Alexandre et al., 2022).

TBRCs have initially been described in LNs supporting the migration and survival of T and B cells at the T–B zone interface through the provision of the B cell–activating factors BAFF and APRIL (Cremasco et al., 2014; Zhang et al., 2018). Expression of the chemokine gene *Ccl19* is reduced in murine LN TBRCs in comparison to TRCs and expression of *Cxcl13* is lower when compared to FDCs and MRCs, which is consistent with their intermediate position between T and B cell zones (Perez-Shibayama et al., 2020; Pikor et al., 2020; Rodda et al., 2018). TBRCs across murine SLOs additionally express *Ccl21a* and *Fmod* expression (Alexandre et al., 2022; Prados et al., 2021; Pikor et al., 2020; Fig. 1 b). TBRCs in human LNs and tonsils exhibit matching topological and functional properties of their murine counterparts with expression of the chemokines *CXCL13*, *CCL19*, and *CXCL12* and the provision of distinct niche factors to immune cells (Lütge et al., 2023; Fig. 1).

The T cell zone of SLOs is supported by TRCs, which share a common transcriptional profile of high *Ccl19* and *Ccl21a* expression across different SLOs (Fig. 1; Lütge et al., 2021). In addition, T cell zones in LNs and the splenic white pulp contain a subset of *Cxcl9*-expressing TRCs (Alexandre et al., 2022; Kapoor et al., 2021; Pezoldt et al., 2018; Rodda et al., 2018); however, the high abundance of interferon-induced gene signatures suggests that these cells represent TRCs in an activated state (Alexandre et al., 2022; Prados et al., 2021). Single-cell transcriptomics analyses of non-hematopoietic cells in human LNs revealed an FRC subset consistent with a TRC signature including expression of *CCL19*, *CCL21*, and *CCL8* (Abe et al., 2022; Kapoor et al., 2021; Fig. 1 b, right panel). FRCs in the human splenic T cell zone were shown by histological analyses to produce CCL21, CD90, MADCAM-1, and ACTA2 (Alexandre et al., 2022; Steiniger et al., 2014; Fig. 1 b), whereas *CCL1*9, *LUM*, and *PTGDS* expression has been suggested as a transcriptional marker combination to identify human tonsillar TRCs (De Martin et al., 2023; Fig. 1 c, right panel).

The perivascular compartment in SLOs is populated by a range of still insufficiently defined FRC populations, which include *Cd34*^+^ stromal cells surrounding large blood vessels in the murine LN medulla (Rodda et al., 2018) and more broadly distributed *Mcam*^+^ *Acta2*^+^ *Pdgfrb*^+^ PRCs (Perez-Shibayama et al., 2020; Rodda et al., 2018; Fig. 1 a). The murine splenic white pulp harbors *Ly6a*^+^ *Itgb1*^+^ *Pdgfra*^+^ PRCs (Cheng et al., 2019) and *Cd34*^+^ *Clec3b*^+^ *Pi16*^+^ adventitial RCs (ARCs; Alexandre et al., 2022; Fig. 1 b). *Cd34*^+^ *Fbln1*^+^ FRCs have been located in the T cell zone perivascular space of murine Peyer’s patches (Prados et al., 2021; Fig. 1 c). A recent single-cell transcriptomics-based study showed that *ACTA2*^+^ *MCAM*^+^ and *PDGFRB*^+^ PRCs are the major FRC subset in human palatine tonsils (De Martin et al., 2023; Fig. 1 c), indicating that the phenotypic variation and location of PRCs along the vascular tree in both mouse and human SLOs need to be defined in more detail. An adventitial “universal” fibroblast progenitor expressing the marker genes *CD34*, *HAS1*, and *PLIN2* has been identified in different human and mouse tissues (Buechler et al., 2021). Single-cell transcriptomics analysis of human and mouse tissues confirms the presence of *PI16*-expressing FRCs in different SLOs (Alexandre et al., 2022; De Martin et al., 2023; Lütge et al., 2023). Spatial transcriptomics analysis of murine LNs revealed that PI16^+^ RCs (PI16-RCs) most likely locate close to the subcapsular sinus and in the medulla (Lütge et al., 2023), while transcriptomic and histological analysis of human tonsillar PI16-RCs reveals their expression of *CD34*, *FBLN1*, and *MFAP5* and localization underneath the reticulated epithelium (Fig. 1 c, right panel).

Additional FRC subsets generate niches that are specific for particular SLOs such as medullary RCs (MedRCs), which form the FRC network in the LN medulla (Huang et al., 2018). Interfollicular RCs (IFRCs) of LNs that occupy the subcapsular region between B cell follicles can be molecularly distinguished through *Hamp2* and *Cavin2* expression (Perez-Shibayama et al., 2020; Pikor et al., 2020; Fig. 1 a). *Cxcl13*^+^ bridging channel RCs (BCRCs), which connect the splenic red pulp to the T cell zone, express *Ccl21a*, *Cxcl12*, and *Il33*, and show higher transcript abundance of *Tnfsf13b* and *Grem1* in comparison to MRCs and TBRCs (Alexandre et al., 2022; Lütge et al., 2023; Fig. 1 b). A cross-organ comparison of *Cxcl13*^+^ FRCs has confirmed that these subsets are unique to their respective SLOs (Lütge et al., 2023), suggesting that organ-specific FRCs reflect an adaptation to the particular tissue anatomy and means of antigen delivery and lymphocyte trafficking.

In sum, the FRC landscape across mouse and human SLOs shows a number of highly conserved subsets in terms of location and molecular identity. Conserved and specialized FRC subsets exhibit a spectrum of functions that provide optimal catering to immune cells and support scalable environments that facilitate optimal interaction of different immune cell populations.

## Conserved FRC niche functions across lymphoid organs

Based on the concept that protective adaptive immunity depends on repetitive units providing immune surveillance per tissue volume (Cohn and Langman, 1990), the number and size of SLOs require adaptation to body size and immune status. Indeed, the number of LNs in mice kept under specific pathogen–free (SPF) conditions is fairly constant with 22 different LNs across the body (Van den Broeck et al., 2006), whereas the abundance of LNs in (non-SPF) humans is highly variable (Bontumasi et al., 2014), for example, with a range of 6–44 LNs in the neck region (Friedman et al., 1999). Inflammatory or tumor conditions can lead to the formation of ectopic LNs in glandular human tissues such as the female breast (Troupis et al., 2012), implying that FRCs in adult mammalian organisms are actively involved in de novo formation and shaping of SLO environments, as they are during embryonic development.

LNs rapidly adapt to lymphocyte influx and proliferation during inflammation by increasing size and generating space for immune cell retention (Kumar et al., 2010). Swift relaxation of the FRC network during LN expansion is mediated via CLEC-2–PDPN interaction, which reduces actomyosin contractility and adjusts membrane tension of FRCs (Horsnell et al., 2022). After an initial phase of relaxation, FRCs sense the resulting strain through actomyosin (Horsnell et al., 2022) and by mechano-coupling to the underlying matrix of interacting cells via the integrin-coupled force-sensitive adaptor Talin and the downstream transcription factor YAP1 (Assen et al., 2022). Compromised force transduction in FRC-specific *Talin1* deficiency causes severe dysregulation in survival and proliferation of the TRC compartment and a loss of network integrity (Assen et al., 2022). In addition to the transduction of mechanical forces, FRCs are equipped to sense mechanical changes in the SLO environments, for example, via the mechanosensitive ion channel Piezo1, which appears to be important for FRC–immune cell communication in Peyer’s patches and the generation of mucosal antibody responses under steady-state conditions (Chang et al., 2019).

The importance of FRC–immune cell interaction during homeostasis and inflammation can be demonstrated through the physical ablation of FRCs in SLOs using the application of diphtheria toxin (DT) in mice expressing the DT receptor in Ccl19-Cre^+^ cells (Ccl19-iDTR; Novkovic et al., 2016). Loss of FRC network connectivity and decreasing FRC density in the T cell zone results in impaired DC–T cell communication and almost complete deficiency in the activation of antiviral CD8^+^ T cells (Novkovic et al., 2016). Consequently, DT-mediated ablation of FRCs in Ccl19-iDTR mice affects the generation of virus-specific B cell responses, most likely through limiting BRC-dependent T–B interaction and restricting the availability of B cell activating factor (BAFF, TNFSF13B; Cremasco et al., 2014). Likewise, depletion of LN FRCs using DT receptor expression directed by the fibroblast activation protein-α promoter in transgenic mice alters immune cell homeostasis and T cell retention in resting LNs (Denton et al., 2014). Loss of the whole FRC body with the lack of the ECM, deprivation of plasma membrane–associated molecules, and regional deficiency in FRC-derived growth and differentiation factors are the main caveats for the analysis of FRC functions based on the DT receptor approach. Faithful elaboration of critical FRC-derived niche factors for maintenance and regulation of immune cell homeostasis therefore relies mainly on the genetic ablation of key molecules in FRCs in combination with in vitro coculture of FRCs and sorted immune cells.

### MRCs generate macrophage/myeloid cell niches

SLOs are situated at junctions of fluid flow to facilitate immune surveillance of bodily surfaces and internal organs. Epithelial surfaces lining the gastrointestinal tract use active transport of antigen-containing liquids via microfold cells into the antigen sampling zone, i.e., into the subepithelial dome region of Peyer’s patches (Prados et al., 2021). Pathogens can be passively transported via the lymph flow from tissues into regional LNs where subcapsular sinus macrophages capture, for example, viruses and facilitate swift presentation to B cells (Junt et al., 2007). Hematogenic spread of pathogens is initially controlled by distinct macrophage populations residing in the marginal zone of the spleen (Müller et al., 2002; Seiler et al., 1997). The key signals that position and maintain myeloid cells in the antigen sampling zones of SLOs are provided by MRCs.

**Figure 2.**
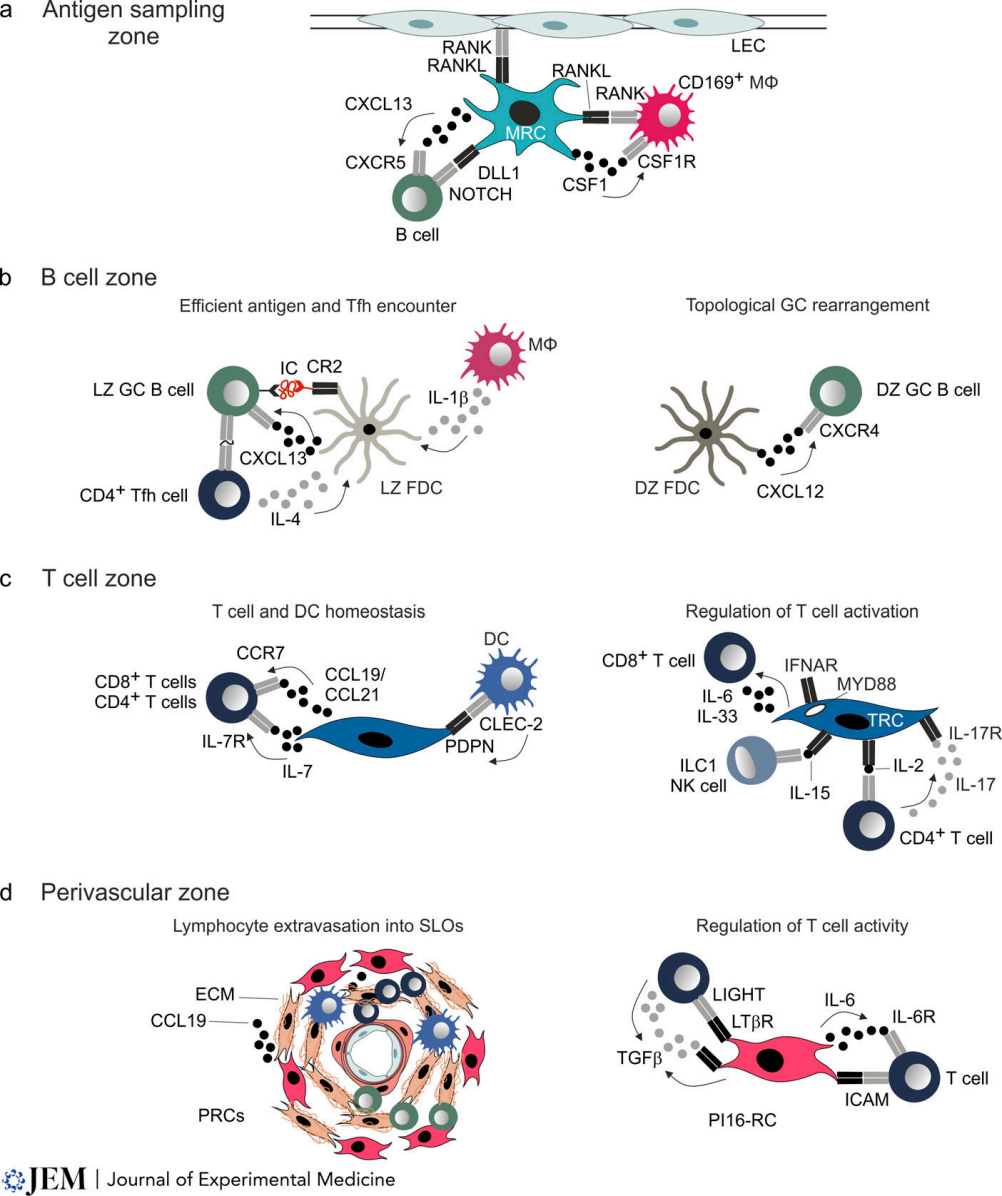
**Molecular interactions between FRCs and leukocytes secure local niche specification and FRC function. (a–d)** Representation of the key molecular signals exchanged between leukocytes and FRC subsets that furnish the antigen-sampling zone (a), B cell zone (b), T cell zone (c), and perivascular zones (d). **(a)** MRCs provided cues to secure niche developments and regulate survival and activation of CD169^+^ macrophages and B cells. **(b)** FDCs integrate and provide diverse cues for efficient antigen and Tfh cell encounter and topological GC rearrangement. **(c)** TRCs provide and receive signals for T cell and DC homeostasis and the regulation of T cell activation. **(d)** In perivascular regions, PRCs and PI16-RCs guide lymphocyte extravasation into SLOs and regulate T cell activity. Arrows indicate the directionality between receptor–ligand interactions. LEC, lymphatic endothelial cell; ECM, extracellular matrix.

Myeloid cells and MRCs form a dense cellular meshwork that underpins either endothelial barriers, i.e., subcapsular sinus floor-lining lymphatic endothelial cells in LNs or vascular endothelial cells in the splenic marginal zone, or an epithelial barrier that overlays the subepithelial dome of Peyer’s patches. Thus, MRCs need to cooperate with other non-hematopoietic cells to generate niches for myeloid cells. Indeed, already during LN development, mesenchymal/fibroblastic FRC progenitors and lymphatic endothelial cells cooperate via the RANK/RANKL axis to shape the sinusoidal macrophage niche (Camara et al., 2019). Genetic ablation of *Rankl* expression in Ccl19-Cre^+^ MRCs resulted in a significant loss of CD169^+^ subcapsular sinus macrophages, reduced retention of viral particles in the subcapsular sinus, and significantly reduced B cell activation following infection with an attenuated vaccinia virus (Camara et al., 2019; Fig. 2 a). In the spleen, expression of the notch ligands delta like 1 (DLL1) by Ccl19-Cre^+^ MRCs is required for the differentiation of marginal zone B cells (Fasnacht et al., 2014; Fig. 2 a). Whether and to what extent MRC-derived DLL1 or other notch ligands affect myeloid cell homeostasis in the splenic marginal zone is, however, still unresolved. In contrast, the contribution of the macrophage master regulator colony-stimulating factor 1 (CSF1, macrophage colony-stimulating factor) for balancing of myeloid cell populations has recently been clarified (Bellomo et al., 2020; D’Rozario et al., 2023; Fig. 2 a). CSF1 is produced by human and mouse LN FRCs and has been shown to stimulate macrophage development and activation in vitro (D’Rozario et al., 2023). In the murine spleen, CSF1 generated by Ccl19-Cre^+^ MRCs appears to be essential for the maintenance of CD169^+^ metallophilic macrophages (Bellomo et al., 2020). Notably, CD169^+^ metallophilic macrophages not only retain blood-borne bacteria and viruses in the splenic antigen sampling zone (Aichele et al., 2003; Seiler et al., 1997) but also directly transfer antigen to antigen-presenting DCs and B cells (Carrasco and Batista, 2007; Gaya et al., 2015). Thus, MRCs in the antigen sampling zone establish a critical niche for myeloid cells that facilitates pathogen recognition, retention, and subsequent initiation of adaptive immune responses.

### BRC-defined niches regulate B cell activation and differentiation

Appropriate formation of the B cell environments is mandatory for the generation of antiviral B cell responses in the LN (Cremasco et al., 2014), protective antibody responses against intestinal helminth infection (Dubey et al., 2016), or control of peritoneal *Salmonella* infection (Perez-Shibayama et al., 2018). Recent studies have elaborated the molecular pathways underlying BRC functions including steering the distribution and availability of antigen, controlling T–B cell interaction, and supporting affinity maturation during the GC reaction.

It has been suggested that the FDC network of the primary B cell follicle is reorganized during inflammatory processes to exclusively underpin the LZ of the GC (Heesters et al., 2014). However, high-resolution single-cell transcriptomics analysis has shown that distinct LZ- and DZ-FDC subsets are predetermined already in the primary B cell follicle and that the poised BRC microenvironments accommodate the GC reaction (Pikor et al., 2020). LZ- and DZ-FDCs undergo transcriptional maturation processes upon inflammation with CXCL12-orchestrated topological FDC remodeling that affects the GC response by limiting extra-follicular B cell responses (Fig. 2 b). CXCL12 chemokine gradients extending past the GC appear to support class-switch recombination at the T–B border through increasing engagement of T cells by activated B cells (Roco et al., 2019). Moreover, the interference with topological FDC remodeling by deleting *Cxcl12* in Cxcl13-Cre^+^ cells attenuates GC responses by limiting the interaction of B cells with T follicular helper (Tfh) cells (Pikor et al., 2020). Thus, poised FDC subset specification and topology establish a feed-forward system that supports the dynamic movement of B cells to engage antigen and cognate T cells and to optimize long-lasting GC responses.

Long-term antigen display by FDCs is a key determinant for the maintenance of protective B cell responses (Bachmann et al., 1996). The structural basis for efficient long-term capture and display by FDCs is underpinned by the high expression of complement and Fc receptors and genes promoting intracellular stiffness and dendrite morphology (Lütge et al., 2023; Pikor et al., 2020). Expression of complement receptor 2 (CR2) by non-hematopoietic cells, most likely FDCs, appears to be particularly important for trapping immune complexes in the GC and maintaining high IgG antibody titers (Fang et al., 1998). Indeed, a recent study by Martínez-Riaño et al. (2023) showed that the topology of CR2 expression in the FDC network of mouse LNs controls antigen retention. Recording of the long-term dynamics of antigen retention in LNs of mice immunized with fluorescent immune complexes and their replacement upon reimmunization revealed that the entire FDC network contributes to initial antigen capture. However, only the central FDCs in the LZ retained antigen and served as long-term antigen reservoirs (Martínez-Riaño et al., 2023). It appears that high concentration or repeated antigen exposure leads to dynamic competition of immune complexes for CR2 (Martínez-Riaño et al., 2023). Indeed, the view on dynamic FDC antigen retention and replacement is compatible with the progressive replacement of naive B cell clones into long-lived GCs during influenza virus or SARS-CoV-2 infection in mice (de Carvalho et al., 2023).

CXCR5- and CXCR4-dependent signals regulate GC B cell and Tfh cell localization in the LZ, where functionally rearranged B cell receptors enable GC B cells to collect antigen and present antigenic peptides to receive help in the form of CD40L-dependent costimulation and cytokines (King et al., 2008). In vitro culture experiments with human LN FDCs have shown that stimulation via the IL-4R increases their CXCL12 production (Pandey et al., 2017), with IL-4 most likely provided in the tissue context by Tfh cells. Indeed, genetic ablation of *Il4ra* expression in murine FDCs suggests that FDC-mediated IL-4 sequestration determines the extent of affinity-matured B cell selection and memory B cell generation (Duan et al., 2021). IL-4 and other cytokines, such as IL-1β, produced by GC-situated macrophages may also directly impact FDC expansion and presentation of immune complexes as shown by in vivo administration of recombinant murine cytokines over the course of a virus-induced GC response (Lütge et al., 2023; Fig. 2 b). Topological BRC disruption by genetic ablation of *Cxcl12* expression in Cxcl13-Cre^+^ cells led to displacement of Tfh cells with reduced cell concentration in the LZ (Pikor et al., 2020), thereby mimicking the impaired GC output and affinity maturation by limiting T cell help. An altered FDC network in the LZ of aged mice appears to be associated with spatial dysregulation of Tfh cell responses and CXCR4-mediated Tfh cell accumulation in the DZ (Silva-Cayetano et al., 2023). FDC expansion and maturation after immunization appear to be limited by hitherto unknown aging-associated processes leading to reduced LZ size and dispersion of Tfh cells throughout the GC, which may be one of the main factors for the reduced quality of the antibody response in aged individuals (Silva-Cayetano et al., 2023). Finally, the finding that CXCL13 production in murine BRC populations is regulated by IL-4 and other FDC-activating factors such as vascular endothelial growth factor-β, progranulin, and transforming growth factor-β1 (TGF-β1), highlights that redundant molecular pathways regulate the establishment of B cell niches (Lütge et al., 2023).

### TRC-dependent control of immune cell interactions in the T cell zone

T cell activation and differentiation are mainly initiated and regulated in the T cell zone by the interaction of different DC populations with T cells (Gasteiger et al., 2016). Migration of naive T cells to the T cell zone is mediated via the TRC-derived chemokines CCL19 and CCL21 (Junt et al., 2002; Link et al., 2007; Fig. 2 c). In addition, TRCs are one of the main sources of IL-7, which has been shown to promote survival of T cell survival in vitro (Link et al., 2007; Fig. 2 c). However, deletion of *Il7* expression in FRCs in *Prx1*-Cre mice appears to leave naive T cell homeostasis unaltered with only moderate reduction of CD8 central memory T cell in peripheral LNs and spleen (Knop et al., 2020). Notably, IL-7 can be provided to T cells by other non-hematopoietic cells such as lymphatic endothelial cells (Onder et al., 2012), highlighting that key immune activation and maintenance circuits are secured by partially overlapping and redundant molecular pathways. Indeed, the ability of TRCs to promote immune responses can be demonstrated by impaired FRC maturation due to selective LTβR deficiency in Ccl19-Cre^+^ cells, which results in reduced TRC function leading to impaired antiviral immunity (Chai et al., 2013). Since FRCs that are located in the T cell zone provide factors that globally attenuate T cell responses (Knoblich et al., 2018; Lukacs-Kornek et al., 2011), TRCs are considered a key cell population that equilibrates T cell reactivity, most likely through the integration of molecular cues from the microenvironment to balance T cell–activating and –attenuating signals.

The finding that FRCs in murine LNs express and display antigenic peptides derived from peripheral tissue antigens (Fletcher et al., 2010) suggests that direct antigen presentation by FRCs affects T cell responses. Indeed, a recent study using genetic ablation of the MHCII molecule in Ccl19-Cre^+^ cells revealed that FRCs shape CD4^+^ T cells’ responses in a murine model of acute graft-versus-host disease (GVHD) with reduced expansion of FoxP3-expressing CD4^+^ T cells and invariant natural killer (NK) T cells (Shaikh et al., 2022). Likewise, ECM components provided by FRCs have been shown to alter the effector–regulatory T cell ratio with increased allograft reactivity in mice lacking laminin α4 expression on Pdgfrb-Cre^+^ cells (Li et al., 2022). Further evidence for the ability of TRCs to regulate T cell responses during infections is shown by the ablation of the innate immunological sensing adaptor MyD88 in Ccl19-Cre^+^ cells in murine Peyer’s patches (Gil-Cruz et al., 2016). Ccl19-Cre^+^ FRCs in Peyer’s patches are located mainly in the T cell zone (Prados et al., 2021), where they directly sense the presence of pathogens resulting in the down-tuning of IL-15 production and reduction of IL-15 trans-presentation (Gil-Cruz et al., 2016). Disabling MyD88-dependent limiting of IL-15 availability in Peyer’s patches resulted in overshooting activation of ILC1 and NK cells, which favored immunopathological T helper 1 and reduced regulatory T cell activity (Gil-Cruz et al., 2016; Fig. 2 c). A second indirect mechanism to regulate T cell reactivity employed by TRCs is the accommodation of different DC subsets in specific niches. For example, FRC-derived DLL1 is not only necessary for splenic marginal zone B cell homeostasis but also affects the development of endothelial cell adhesion molecule-expressing DCs, which together foster Tfh cell activation and immune responses against the parasite *Schistosoma mansoni* (Fasnacht et al., 2014). Similarly, *Grem1*-expressing FRCs in murine LNs, which are mainly located at the T–B border, generate the niche environment for pre-DC and conventional DC populations (Kapoor et al., 2021). Physical ablation of *Grem1*^+^ TRCs results in decreased homeostatic proliferation and survival of DCs (Kapoor et al., 2021), which compromises T cell immunity through still-unknown mechanisms.

Utilization of FRC-specific ablation of key molecules has revealed a number of mechanisms of TRC-mediated control of T cell function including trans-presentation of IL-2 via CD25 (Kim et al., 2022; Fig. 2 c). The absence of CD25 on TRCs altered T cell differentiation by promoting the phenotype of T helper 17 (Th17) cells (Kim et al., 2022). A further molecular circuit connecting FRC and T cell activation is steered by the type I interferon receptor (IFNAR). Viral infection of LN FRCs by the lymphocytic choriomeningitis virus (LCMV) leads to global FRC activation with a dominant pattern of interferon-induced gene activity, which is important to halt viral replication in LNs and to limit systemic spread (Perez-Shibayama et al., 2020). Moreover, *Ifnar* deficiency of Ccl19-Cre^+^ cells fosters exhaustive CD8^+^ T cell activation and thus reduces protective antiviral T cell immunity (Perez-Shibayama et al., 2020; Fig. 2 c). It appears that the effects of IFNAR signaling in FRCs depends on the nature of the viral infection as shown by the fact that genetic *Ifnar* deletion in Prx1-Cre^+^ cells alters CD8^+^ memory responses following infection with the cytopathic vesicular stomatitis virus (Knop et al., 2022). Clearly, the provision of a broad range of effector molecules, including IL-33, by activated FRCs is an important step in the generation of antiviral immune responses. Genetic ablation of *Il33* expression in Ccl19-Cre^+^ cells results in the reduction of virus-specific CD8^+^ T cells that produce IFN-γ and TNF during LCMV clone 13 infection (Aparicio-Domingo et al., 2021). Moreover, the production of innate immunostimulatory molecules such as IL-6 that are produced during the acute phase of viral infection outweigh negative regulatory effects of nitric oxide synthase 2–dependent production of nitric oxide (Lukacs-Kornek et al., 2011), augmenting virus-specific CD8^+^ T cell memory responses including differentiation into tissue-resident memory T cells (Brown et al., 2019). In sum, TRCs generate important niches in the T cell zone where they directly interact with T cells and foster the interaction of T cells with DCs and other immune cells. It appears that the outcome of such direct and indirect intercellular communication is determined by the context and duration of particular perturbations (e.g., acute versus chronic infection/inflammation). It has to be highlighted here that the mentioned murine models for FRC targeting are not TRC specific. The assignment of the specific functions for TRCs is therefore largely based on the assumption that T cell activation and differentiation are exclusively controlled in the T cell zone. Future efforts should thus be steered towards the development of model systems that facilitate FRC subset-specific genetic targeting.

### Immune cell niches underpinned by PRCs and PI16-RCs

The blood vasculature is a structural key component of SLOs with functions ranging from the provision of oxygen concentration in SLO compartments (Huang et al., 2007), balancing fluid content (Acton et al., 2021), to the regulation of immune cell immigration (Ager, 2017). Perivascular fibroblastic cells support blood endothelial cells through the generation of a basement membrane, regulate blood vessel contractility, and provide immune cell niches in proximity to vessels. The functional properties, morphological appearance, and transcriptional profile of perivascular cells depend on the concentric distance to the blood vessel and the localization along the vascular tree with pericytes covering capillaries and vascular smooth muscle cells forming contractile layers around arteries and veins (Vanlandewijck et al., 2018). PRCs connect the BRC and TRC networks with the blood vasculature and funnel extracellular liquids from the “pipe-like” conduits in T and B cell zones to perivascular conduit sheaths (Kelch et al., 2019; Novkovic et al., 2020). This connection most likely facilitates rapid transfer of small molecules (Acton et al., 2021) and even larger antibodies (Thierry et al., 2018) from the LN parenchyma to blood circulation. It is interesting to note that PRCs in human SLOs such as the tonsils form a large part of the FRC landscape (De Martin et al., 2023), suggesting that pervasion of SLOs with larger blood vessels—as present in human SLOs—requires structural and functional adaptation in the perivascular space.

A recent study using intravital imaging has shown that immigration of both T and B cells into the murine popliteal LNs includes migration through the endothelial cell barrier followed by crawling in the perivascular conduit sheath and migrating through the PRC network (Choe et al., 2021). Migration across the first PRC layers appears to require particular ECM components (Choe et al., 2021; Fig. 2 d). The finding that migration through the PRC network is most frequent in HEV areas with a higher density of CD11c^+^ DCs (Choe et al., 2021) suggests that PRCs coordinate the interaction of different immune cell populations. Likewise, in the murine spleen, entry of naive T cells from the circulation into the T cell zones of the white pulp is supported by a layer of CCL19-expressing PRCs around arterioles stretching across the bridging channels (Chauveau et al., 2020). CCR7-dependent migration along perivascular T cell tracks is reduced during inflammation (Chauveau et al., 2020), suggesting that rapid remodeling of PRCs in response to inflammatory stimuli contributes to the regulation of intrasplenic immune cell trafficking (Fig. 2 d). However, such phenotypical adaptations of splenic PRCs and the involvement of the recently described BCRCs (Alexandre et al., 2022) in these processes require further investigation.

As predicted from the broad expression of *PI16* in human and mouse tissues (Buechler et al., 2021), PI16-RCs can be detected in murine and human SLOs (Lütge et al., 2023). The findings that PI16-RCs are not represented in pediatric tonsils, that the fraction of PI16-RCs in adult tonsils increases with age and inflammation, and that this specific FRC subset forms an inducible subepithelial compartment with mixed immune cell content suggest that PI16-RCs mainly promote inflammatory processes (De Martin et al., 2023). Indeed, PI16-RCs employ a high diversity of immune cell–activating and ECM remodeling pathways to shape the subepithelial immune cell niche in human tonsils. Conversely, immune cell-derived signals such as tumor necrosis factor superfamily member 14 (TNFSF14) and TGF-β3 most likely contribute to PI16-RC subset specification and activation under inflammatory conditions (De Martin et al., 2023; Fig. 2 d). In line with the immune-activating function of tonsillar PI16-RCs, this particular FRC subset uses canonical immune cell–stimulating pathways such as the proinflammatory cytokine IL-6 or the adhesion molecule ICAM1 to interact with T cells in murine SLOs (Lütge et al., 2023; Fig. 2 d). Thus, PI16-RCs establish an immune-adaptive niche that shapes the microenvironment for immune cell activation and interaction.

Taken together, the perivascular FRC compartment in SLOs supports vascular integrity and regulates immune cell trafficking and activation. However, it is still not clear how PI16-RCs in SLOs relate to PRCs and whether they are strictly located in the adventitial space, as suggested in the initial description of PI16-expressing fibroblasts (Buechler et al., 2021).

## FRC–immune cell interactions in inflammatory diseases and cancer

The FRC niche concept outlined above reflects the dynamic regulation of FRC phenotype and function. Accordingly, FRCs serve as “caterers” to immune cells during homeostasis and inflammation through the provision of migration, growth, and differentiation cues that are key for the regulation of protective immunity during infection. Conversely, adverse FRC remodeling can foster immunopathological inflammation or support establishment of tumor-supportive niches in SLOs.

### FRC activation in autoimmune and inflammatory disorders

As in antiviral immunity, the modulation of T cell differentiation by FRCs appears to determine the activation and differentiation of T cells in chronic inflammatory diseases. In allogenic bone marrow transplantation, the provision of Notch ligands to donor T cells is critical to trigger GVHD. In the splenic white pulp, CD157^+^ FRCs and FDCs appear to be the critical providers of these ligands to alloimmune T cells (Chung et al., 2017). FRC-specific deletion of DLL1/4 in Ccl19-Cre^+^ FRCs abrogates the production of pathogenic cytokines and expression of gut-homing integrins by alloimmune T cells, limiting intestinal inflammation and preventing GVHD (Chung et al., 2017; Tkachev et al., 2023). Additionally, early blockade of Notch, within the first 48 h following transfer, permits the expansion of donor FoxP3^+^ regulatory T cells (Chung et al., 2017). The ability of donor T cells to recognize allo-MHC-peptide complexes presented by Ccl19-Cre^+^ FRCs appears to further license the expansion of regulatory T cells and attenuate the severity of GVHD (Shaikh et al., 2022). Moreover, CD25-mediated IL-2 trans-presentation by LN FRCs attenuates Th17 cell differentiation resulting in dampened tissue pathology in Th17-mediated autoimmune disorders such as experimental autoimmune encephalomyelitis, experimental psoriasis, and antigen-induced arthritis (Kim et al., 2022). Thus, lymphoid organ FRCs steer pathogenic T cell activation and consequent immunopathology.

In addition to modulating T cell differentiation, FRCs appear to integrate signals from recently activated T cells. For instance, provision of IL-17 by locally differentiating Th17 cells mediates FRC metabolic reprogramming in inflamed LNs and spleen. Lack of IL-17 receptor signaling in Ccl19-Cre^+^ FRCs leads to cell cycle arrest and apoptosis, impaired proliferation with decreased *Fn1*, *Col1a1*, and *Col3a1* expression, compromised GC formation and antibody production (Majumder et al., 2019; Fig. 2 c). Notably, inflammation-remodeled FRCs appear to maintain epigenetic imprints and enhanced metabolic status following an initial inflammatory trigger, altering the protective antibody-mediated immunity and colonic immunopathology during subsequent infections (Wu et al., 2021). Similarly, mesenteric LN and Peyer’s patch FRCs dampen ILC1 and NK cell activation and secure intestinal barrier integrity following enteric viral infection (Gil-Cruz et al., 2016). Thus, FRCs can initiate inflammatory reactions by direct sensing of pathogenic material via TLRs and cell stress–mediated signals or they can enter an inflammatory mode integrating immune cell–derived signals such as cytokines. In either case, stimuli provided by FRCs crucially contribute to optimal immune cell activation to maintain the balance between immunity and immunopathology.

### FRC remodeling in cancer

Metastatic seeding of LNs represents a critical step toward the spread of tumor cells within the body. First, studies shedding light on tumor-draining LN FRCs indicate that tumor cell–produced factors induce a premetastatic FRC reprogramming that favors tumor invasion of the LN niche. In preclinical melanoma models, enlargement of tumor-draining LNs preceding metastasis is associated with an increased number and gradual reprogramming of key signaling pathways of PDPN^+^ FRCs (Riedel et al., 2016). Activated FRCs exhibit increased oxidative phosphorylation, *Pdpn* and *Thy1* upregulation concomitant with downregulated expression of *Il7* and *Ccl21*, and increased expression of genes encoding for ECM components and microtubule cytoskeleton rearrangement. In line with such phenotypic changes in melanoma tumor-draining LN FRCs, oxidative phosphorylation programs are upregulated in tumor-draining LN FRCs in a metastatic breast cancer model (Li et al., 2020), and an inverse regulation of FRC PDPN and CCL21 expressions are observed in murine and in vitro B cell lymphoma models (Apollonio et al., 2023). Similarly, metastatic LNs from patients with invasive breast cancer display a partial or complete loss of CCL21 in perivascular ACTA2^+^ FRC of highly dilated HEVs (Bekkhus et al., 2021). Such tumor-induced remodeling of FRCs, in particular, reduced CCL21 expression, is associated with perturbed lymphocyte compartmentalization and proliferation (Riedel et al., 2016), while dampened IL-7 production is associated with reduced T cell numbers (Gao et al., 2017). Nevertheless, to what extent premetastatic FRC reprogramming through described and yet-unknown factors affects immune cell recruitment, function, and disease outcome in different tumor types remains subject to further studies.

The phenotypical and structural remodeling of the PDPN^+^ FRC network appears to be driven by tumor-derived factors. Lactic acid, a metabolite released by cancer cells and found to be enriched in tumor-draining LNs of preclinical melanoma and breast cancer models, was identified to modulate FRC metabolism and mitochondrial function, leading to FRC activation as marked by upregulation of PDPN and Thy1 and downregulation of IL-7, without affecting survival and proliferation of FRCs (Riedel et al., 2022). Furthermore, soluble factors released by dedifferentiated melanomas are found to promote FRC elongation, proliferation, and upregulation of the activation markers PDPN, tenascin C, fibronectin, and IL6 in vitro (Rovera et al., 2022). Among the factors secreted by dedifferentiated melanoma cells, IL1α and IL1β cytokines inhibit the contractility of primary human LN fibroblasts and thereby enhance cancer cell invasiveness in vitro. While premetastatic LN FRC activation appears to be driven by tumor-derived factors that drain from the malignant tissue, additional contact-dependent, tumor-derived factors may orchestrate FRC remodeling in metastatic LNs (Apollonio et al., 2023).

Much less is known about the molecular mechanisms mediating FRC reprograming in tumor-draining LNs or lymphoma-bearing lymphoid tissues. FRC relaxation appears to be driven by impaired JAK1-Stat3 signaling, decreased RhoA-Rho pathway activity, and cytoplasmic translocation of the mechano-receptor YAP1 (Rovera et al., 2022), with TNFR and LTβR signaling contributing to the elongation of FRCs in vitro (Apollonio et al., 2023). Bulk RNA sequencing and histological assessment of high-tumor-burden follicular lymphoma tissues demonstrate a significant correlation between *LTB*, *TGFB1*, and *CCL21* expression (Mourcin et al., 2021). While TNF and LTβR signals are required to induce cytokine production by follicular lymphoma FRCs in vitro, the largest additive effect is driven in combination with TGFβ (Mourcin et al., 2021). To date, our understanding of the key signals driving FRC remodeling relies primarily on in vitro experiments, where changes in fibroblast morphology may be especially susceptible to FRC removal from their tissue environment. Transcriptomic and histological validations of patient tissues are needed to strengthen in vitro findings, and genetic models perturbing key receptors and transcriptional regulators in distinct FRC subsets are critical to delineate the underlying mechanisms steering FRC remodeling and the impact on antitumor immunity.

## Conclusions and future research directions

FRCs physically underpin and functionally support protective immune responses by establishing four conserved major regions in SLOs, i.e., antigen-sampling zone, B cell zone, T cell zone, and the perivascular compartment. The view of repetitive and scalable FRC environments in the different SLOs is supported by the presence of highly conserved FRC subsets in equivalent locations and sharing molecular identity. In addition, SLO-specific milieu factors facilitate optimal catering to immune cells and support interaction of different immune cell populations. Single-cell transcriptomics-based analyses have generated a high-dimensional view on the FRC landscape in murine and human SLOs with the identification of multiple intracellular and surface markers for conserved and SLO-specific FRC subsets. Future research efforts will need to focus on the validation of markers and marker combinations that allow for FRC subset distinction in histological sections and by flow cytometry. Validated markers that signify FRC subsets in their homeostatic and activated state will be instrumental to establish diagnosis and prognosis for different disease conditions including chronic infection, autoimmunity, and cancer.

The core position of FRCs in the SLO environment is based on their ability to respond to a broad range of stimuli that are recognized by different receptors including mechano-receptors that detect changes in tissue tension and stiffness, surface receptors that bind cytokine, growth, and differentiation factors, and intracellular receptors. FRCs integrate these highly diverse signals in a context-specific manner to adapt and optimize immune cell interaction in the different niche environment contributing to either activating or regulating overall conditions. Comprehensive analysis of FRC subset functions therefore needs to take into account FRC subset identity, tissue context, and overall inflammatory conditions including the composition and activation status of interacting immune cells. FRC-specific genetic ablation of key molecules via the Cre-loxP system in combination with defined perturbation (e.g., viral infection, induction of autoimmune disease, or tumor challenge) has been instrumental to better define changes in FRC phenotype and functional properties in murine models. Further development of this approach, for example, through the combination of Cre and Dre recombinases (Weng et al., 2022), could increase the precision of FRC subset targeting. Elaborating the function of human FRC subsets and interaction patterns with immune cells is mainly based on in vitro (co-)culture systems that could be further improved by adopting the latest organoid and microfluidics technology to better emulate the tissue context including migration patterns of autologous immune cells.

Tumor-induced remodeling of FRCs in premetastatic and tumor-bearing lymphoid tissues is widely associated with immune-suppressive changes in leukocyte compartmentalization and function. FRC reprogramming may also promote tumor growth by promoting myeloid cell differentiation and polarization. Targeting tumor-derived cues to impede cancer cell–supportive FRC reprogramming could be an effective strategy to prevent malignant LN colonization and spread of disease. However, to what extent premetastatic FRC reprogramming through described and yet-unknown factors affects immune cell recruitment and function and disease outcome in different tumor types remains subject to further studies.
